# Clinical rarity: simultaneous choroid plexus papilloma and extraventricular neurocytoma presenting as intraventricular hemorrhage in an adolescent

**DOI:** 10.1093/jscr/rjad699

**Published:** 2024-01-11

**Authors:** Kaleb Derouen, Kierany Shelvin, Wesley Shoap, Randall Craver, Jerome Volk, Oritsejolomi A Roberts

**Affiliations:** Department of Neurosurgery, Children’s Hospital of New Orleans, New Orleans, LA, United States; Department of Neurosurgery, Children’s Hospital of New Orleans, New Orleans, LA, United States; Department of Neurosurgery, Children’s Hospital of New Orleans, New Orleans, LA, United States; Department of Neurosurgery, Children’s Hospital of New Orleans, New Orleans, LA, United States; Department of Neurosurgery, Children’s Hospital of New Orleans, New Orleans, LA, United States; Department of Neurosurgery, Children’s Hospital of New Orleans, New Orleans, LA, United States

**Keywords:** pediatric neurosurgery, choroid plexus papilloma, neurosurgery

## Abstract

We present a patient with an intraventricular hemorrhage. Imaging identified a left atrial intraventricular mass and a vague adjacent second periventricular cystic lesion. A guided trans-sulcal approach via a left parietal craniotomy resulted in a gross total resection of both lesions. These represented two distinct lesions, the periventricular cystic lesion was an extraventricular neurocytoma (EVN) and a World Health Organization grade 1 choroid plexus papilloma (CPP). The neurocytoma required methylation studies for confirmatory diagnosis. The patient had an uneventful recovery with a normal neurological exam at 12-weeks. This documents the occurrence of two distinct central nervous system tumors, a CPP and an EVN presenting with an intraventricular hemorrhage.

## Introduction

The choroid plexus, a complex network of neuroepithelial cells situated within the cerebral ventricles, plays a critical role in the production and secretion of cerebrospinal fluid [1]. Choroid plexus neoplasms manifest in three distinct forms, distinguished by their World Health Organization (WHO) grading: papilloma (WHO grade 1), atypical (WHO grade 2), and carcinoma (WHO grade 3). Choroid plexus tumors represent a subset of brain malignancies, with an annual incidence of merely 0.3 cases per million [2]. From a comprehensive meta-analysis, the median age of diagnosis is 3.5 years [2]. In children, these tumors predominantly arise in the lateral ventricles, while in adults, their primary site is the fourth ventricle [3]. While traditionally considered sporadic, emerging evidence has demonstrated associations between choroid plexus papilloma (CPP) and various genetic conditions, such as Li-Fraumeni syndrome and Aicardi syndrome [4]. Diagnosis routinely relies on a battery of imaging modalities, including neurosonography, computed tomography (CT) scans, and magnetic resonance image (MRI), aimed at characterizing tumor location, dimensions, and suitability for surgical intervention. Presently, the first-line therapeutic approach for CPP is surgical resection, with documented recurrence frequencies being low, with cures approaching 100%.

Central neurocytomas are often located within the ventricular cavities, and the less frequent extraventricular neurocytomas (EVN) can be located throughout the central nervous system. In 2007, WHO officially classified EVN as a distinct tumor subtype, separating it from central neurocytomas [5]. Beyond their differing anatomical locations, these two variants also display contrasting histological characteristics, with EVNs typically presenting as less cellular and exhibiting a more heterogeneous histological appearance [6]. Given the limited number of reported cases, the literature surrounding EVNs remains scant, offering limited insights into risk factors, clinical manifestations, diagnostic strategies, and treatment modalities [7]. EVNs have no pathognomonic radiographic signs, and often mimic other astrocytic glial tumors [8, 9]. Due to their extremely low incidence, there is no established consensus on the treatment of EVNs [9]. Nevertheless, current literature suggests that complete tumor resection is considered the optimal treatment approach [10, 11].

## Case illustration

**Figure 1:**
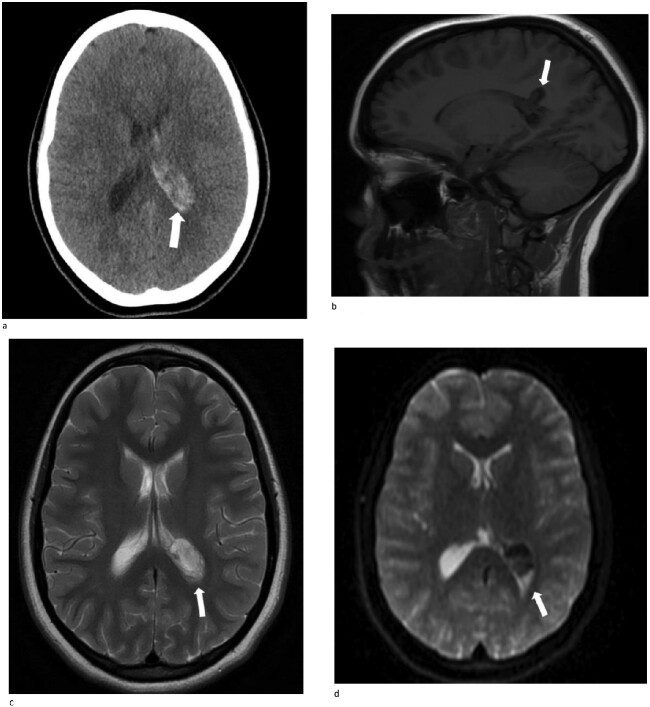
(a)–(d) Demonstrates evidence of a hemorrhagic lesion involving the left atrium and adjacent periventricular deep white matter with a moderate amount of intraventricular hemorrhage within the left ventricle.

A 13-year-old female presented with an abrupt onset of a severe headache accompanied by two episodes of vomiting, left-sided jaw pain, nausea, and nasal congestion. She did not report any numbness, tingling, muscle weakness, or changes in vision. An initial non-contrast CT scan of the head revealed a left lateral intraventricular hemorrhage, (Fig. 1a). There were no intraparenchymal hemorrhages, midline shifts, or extra-axial fluid accumulations. MRI revealed a hemorrhagic lesion measuring 2.1 cm × 1.6 cm × 2.6 cm, involving the left atrium and the adjacent periventricular deep white matter (Fig. 1b). There was thickening of the choroid plexus, with no significant vasogenic edema or acute extra-axial fluid collection, and no signs of brain herniation.

On neurological exam, she exhibited intact cognition and appropriate mental status. Pupils were equal, round, and reactive to light. She experienced pain upon extraocular movements. There were no discernible cranial nerve deficits, sensory abnormalities, motor weakness, and her gait was within normal parameters.

The child underwent right frontal external ventricular drain placement. The following day, a cerebral angiogram suggested the presence of a left intraventricular mass. The angiogram revealed a 9 mm × 15 mm left choroidal plexus tumor blush, located in the posterior aspect of the choroid plexus, with no arteriovenous fistulas or arteriovenous malformations. Nevertheless, it remained unclear whether two separate lesions were present.

A left parietal craniotomy with neuro-monitoring, stereotactic guidance, and intraoperative ultrasound resected both lesions. A “U” shaped dural opening was created, oriented toward the sagittal sinus, allowing for the identification of the primary motor and sensory cortices. Guided dissection enabled access to the atrium of the lateral ventricle, where the choroid plexus had a globe shape tumor. The adjacent cystic tumor was also accessed by this approach. Both lesions were completely removed. There were no intraoperative complications.

### Pathology

**Figure 2:**
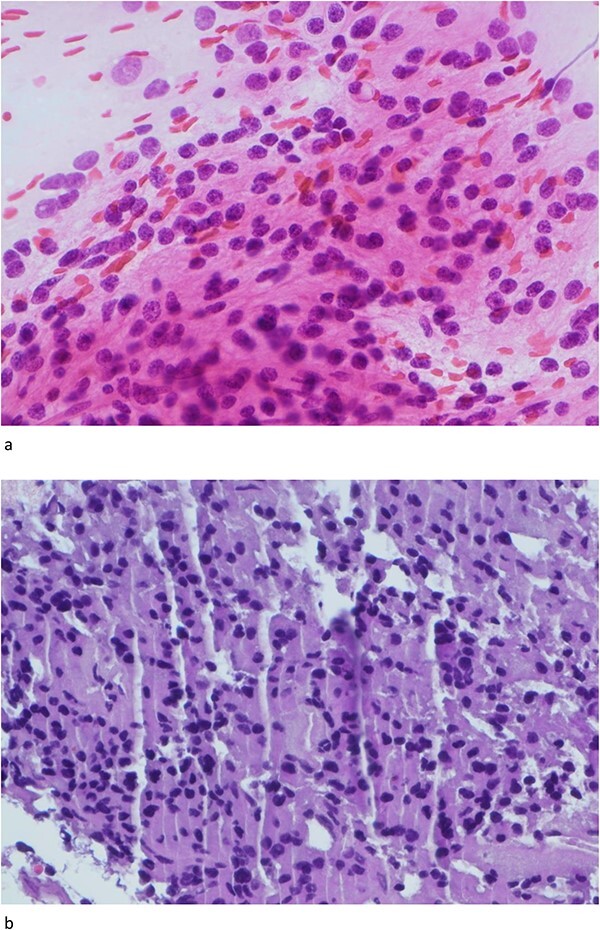
(a) and (b) The touch preparation and frozen section control shows similar histologic features. The cystic lesion shows a moderately hypercellular tumor, with uniform round nuclei, irregular distribution of chromatin, and a moderate amount of eosinophilic cytoplasm. There is no distinct fibrillary background (Hematoxylin and eosin, 400×).

**Figure 3:**
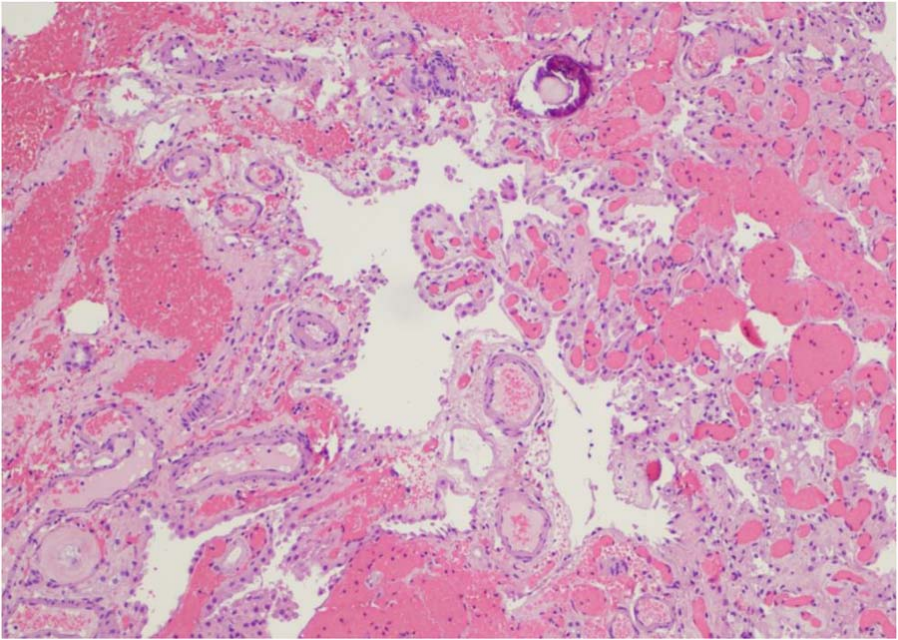
The intraventricular tumor consisted cuboidal epithelium overlying vascularized papillae. The nuclei are uniform without atypia. A psammomatous calcification is present in the upper portion of the field (Hematoxylin and eosin, 100×).

**Figure 4:**
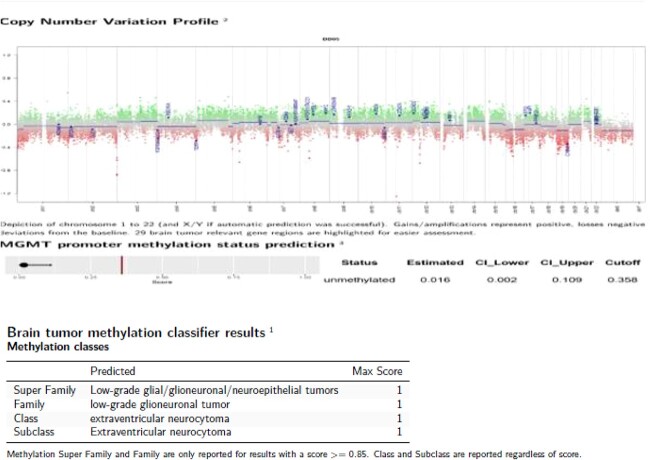
Methylation study results.

Only one small piece of the cystic lesion was obtained and entirely submitted for touch preparation and frozen section (Fig. 2), which demonstrated a hypercellular lesion composed of uniform ovoid cells with salt-and-pepper chromatin, lacking pleomorphism, grooves, nucleoli, increased mitotic activity, and had no papillary architecture. The intraventricular lesion was submitted separately and had the morphology of a benign papilloma (Fig. 3), staining with cytokeratin AE1/AE3, with no staining with GFAP. Because of the histological discrepancy of the first and second specimens, and the limited amount of tissue from the cystic lesion, the paraffin embedded tissue of the cystic lesion was submitted for methylation profiling by NIH. The methylation profiling had a maximum score of 1 for both class and subclass for EVN, WHO grade 2 (Fig. 4).

### Post-operative course

**Figure 5:**
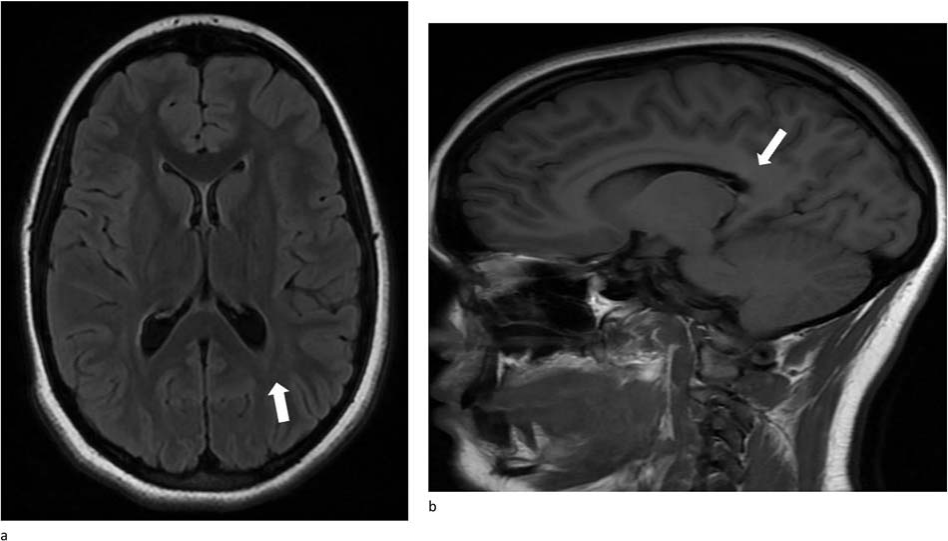
(a) and (b) Stable postsurgical findings compatible with gross total resection of left atrial and adjacent periatrial tumor. No MRI evidence of residual/recurrent mass, with normal ventricular size.

At her 12-week post-operative evaluation, follow-up MRI scans showed no residual or recurrent disease, as illustrated in Fig. 5. This patient’s genetic workup has demonstrated no propensity for neoplastic disease so far and she has resumed customary activities and routines.

However, it is worth noting that early in her post-operative course she did encounter challenges related to visual acuity. She has since been receiving care by an ophthalmologist and now utilizes corrective glasses to manage her visual concerns. She continued to have intact cranial nerve function and 5/5 bilateral strength post-operatively.

## Discussion

Choroid plexus tumors account for ˂1% of all reported cases, with a slightly higher frequency observed in the pediatric population, where they manifest in ~3–4% of cases [10]. The precise frequency of EVN in children remains unknown. Despite their benign nature, both CPPs and EVNs can result in significant morbidity, often associated with heightened intracranial pressure, and the development of hydrocephalus. Consequently, patients with these tumors typically present with a spectrum of symptoms, including nausea, vomiting, visual disturbances, unsteady gait, and pulsatile headaches [11].

Our patient’s presentation was consistent with expectations and characterized by a sudden-onset severe headache. To the best of our knowledge, no prior documented case in the existing literature has reported the simultaneous presentation of these two distinct tumor types culminating in intraventricular hemorrhage. The occurrence of intraventricular hemorrhage in an adolescent patient is a rare clinical finding. Awareness of the varied and often unusual clinical manifestations of pediatric brain tumors enables prompt recognition and targeted intervention for this complex pathology.

Existing literature underscores that due to their benign character, CPPs and EVNs can be effectively cured through gross total resection. Surgical resection of these tumors can pose formidable challenges, attributable to their anatomical location, size, marked vascularity, and the relatively small blood volume in young patients [11].

This case report contributes to the limited body of literature addressing the management of co-occurring benign tumors, specifically CPP (WHO grade 1) and EVN (WHO grade 2). Therefore, we believe that the data presented in our single-center case report can enhance our understanding of these exceedingly rare tumors. This contribution is valuable not only for the existing literature but also for future meta-analyses of this medical condition.

## Author contributions

KD analyzed and interpreted the data and was the major contributor in writing the manuscript. All other authors reviewed and approved the manuscript.

## Data Availability

The authors declare that all of the data is available within this article.
